# Evaluation of biocompatibility of novel and commonly-used antiseptics by cell culture method

**DOI:** 10.1186/2047-2994-4-S1-P38

**Published:** 2015-06-16

**Authors:** M Yamamoto, R Matsumura, Y Hirata, H Nagamune

**Affiliations:** 1 Department of Biological Science and Technology, Institute of Technology and Science, Tokushima University Graduate School, Tokushima, Japan; 2 Biochemical Laboratory, SARAYA CO. LTD., Osaka, Japan

## Introduction

Antiseptic agents such as chlorhexidine digluconate, polyhexamethylene biguanide, benzalkonium chloride and octenidine dihydrochloride (octenidine) has been used widely in clinical settings to prevent bacterial infection, especially for antisepsis of skin and mucous membranes. However, irritation and cytotoxic effects of these compounds on human cell has been reported.

## Objectives

This study compared the biocompatibility of newly-synthesized bis-quaternary ammonium compounds and biocides that have been commonly used as antiseptics, examining their antimicrobial activity, cytotoxic effect and expression of inflammatory cytokine-related genes.

## Methods

A series of new bis-quaternary ammonium compounds, including *3-*(*3-Hydroxy-2-*(*hydroxymethyl*)*-2-{[*(*1-dodecylpyridinium-3-yl*)*oxy]methyl}propoxy*)*-1- dodecylpyridinium dibromide* (3HHDMP) was synthesized.The antimicrobial activity of newly-synthesized compounds and commonly-used antiseptics were assessed by minimum bactericidal concentrations (MBC) and cytotoxic effect on normal human epidermal keratinocytes.The expression of inflammatory cytokines in normal human epidermal keratinocytes was quantified using real-time reverse transcription PCR (RT-PCR).

## Results

Against Gram-negative and Gram-positive bacteria, 3HHDMP showed potent antimicrobial activity comparable to that of the octenidine. In a cytotoxic test using human epidermal keratinocytes, toxicity of 3HHDMP was equal or lower compared to that of quaternary ammonium compounds, although 3HHDMP showed higher toxicity than biguanide-based compounds. The comparison of the biocompatibility index as defined by antimicrobial activity and cytotoxic effect on human cell revealed that 3HHDMP had equal or greater biocompatibility compared with the biocides tested. Moreover, in the expression analysis of cytokine-related genes by cell culture method, increase of expression of cytokine-related genes in the cells stimulated with 3HHDMP was slower than that of existing quaternary ammonium compounds.

## Conclusion

From these results, it was concluded that the series of new bis-quaternary ammonium compounds including 3HHDMP had excellent biocompatibility, and thus the availability for application to the skin surface was suggested.

## Disclosure of interest

None declared.

